# Stereotactic body radiation therapy with or without transarterial chemoembolization for patients with primary hepatocellular carcinoma: preliminary analysis

**DOI:** 10.1186/1471-2407-8-351

**Published:** 2008-11-27

**Authors:** Byung Ock Choi, Ihl Bohng Choi, Hong Seok Jang, Young Nam Kang, Ji Sun Jang, Si Hyun Bae, Seung Kew Yoon, Gyu Young Chai, Ki Mun Kang

**Affiliations:** 1 Department of Radiation Oncology, The Catholic University of Korea, School of Medicine, Seoul, South Korea; 2 Department of Internal Medicine, The Catholic University of Korea, School of Medicine, WHO Collaborating College of Medicine, Seoul, South Korea; 3 Department of Radiation Oncology, Gyeongsang National University, College of Medicine, Gyeongsang Institute of Health Sciences, Jinju, South Korea

## Abstract

**Background:**

The objectives of this retrospective study was to evaluate the efficacy of stereotactic body radiation therapy (SBRT) for small non-resectable hepatocellular carcinoma (HCC) and SBRT combined with transarterial chemoembolization (TACE) for advanced HCC with portal vein tumor thrombosis (PVTT).

**Methods:**

Thirty one patients with HCC who were treated with SBRT were used for the study. We studied 32 HCC lesions, where 23 lesions (22 patients) were treated targeting small non-resectable primary HCC, and 9 lesions (9 patients) targeting PVTT using the Cyberknife. All the 9 patients targeting PVTT received TACE for the advanced HCC. Tumor volume was 3.6–57.3 cc (median, 25.2 cc) and SBRT dose was 30–39 Gy (median, 36 Gy) in 3 fractions for consecutive days for 70–85% of the planned target volume.

**Results:**

The median follow up was 10.5 months. The overall response rate was 71.9% [small HCC: 82.6% (19/23), advanced HCC with PVTT: 44.4% (4/9)], with the complete and partial response rates of 31.3% [small HCC: 26.1% (6/23), advanced HCC with PVTT: 11.1% (1/9)], and 50.0% [small HCC: 56.5% (13/23), advanced HCC with PVTT: 33.3% (3/9)], respectively. The median survival period of small HCC and advanced HCC with PVTT patients was 12 months and 8 months, respectively. No patient experienced Grade 4 toxicity.

**Conclusion:**

SBRT for small HCC and SBRT combined with TACE for advanced HCC with PVTT showed feasible treatment modalities with minimal side effects in selected patients with primary HCC.

## Background

Primary hepatocellular carcinoma (HCC), which comprises 90% of all malignant cancers developed in the liver, is a fatal disease that might cause death with severe complications unless treated properly [1,2]. Many modalities such as surgical resection, percutaneous ethanol injection (PEI), radiofrequency ablation (RFA), Y90 microspheres, external radiation therapy (RT) and transarterial chemoembolization (TACE) have been tried in the treatment for HCC [3-8], but the optimal treatment approach remains controversial.

Stereotactic radiation therapy (SRT), for benign and malignant diseases, was initially used only for intracranial lesions. With the advent of advanced imaging techniques and robotic image-guided radiation technologies, the Cyberknife has extended highly conformal radiosurgery to extracranial SRT applications [9]. Stereotactic body radiation therapy (SBRT) is now being extended to more patients and clinical targets [10,11]. To date, there are only a few reports in the literature that assessed the response of HCC to SBRT [12-16]. We have employed SBRT, using the LINAC-based SBRT since 1995, with a special focus on liver tumors [17,18]. We performed LINAC-based SBRT for 20 primary HCC patients with the result of 80% local control, thus confirming that SBRT is helpful in the treating primary HCC.

Expanding our experience further, we have attempted SBRT alone for small primary non-resectable HCC, and used the combined therapy of TACE and SBRT for advanced HCC with portal vein tumor thrombosis (PVTT). Therefore, we evaluated the response rate and toxicity of SBRT for small primary non-resectable HCC and SBRT combined with TACE for advanced HCC with PVTT.

## Methods

### Patients eligibility

From March 2004 to March 2005, 31 patients participated in a retrospective study at the Cyberknife center, Catholic University. We treated 32 HCC lesions with the Cyberknife (Accuray Inc, Sunnyvale, CA) SBRT: 23 lesions (22 patients) of small non-resectable primary HCC were treated by SBRT while 9 lesions (9 patients) of PVTT in advanced HCCs were treated by SBRT combined with TACE. The criteria for patients to be included in the study were as follows: (1) patients with histologically proven primary HCC by ultrasound guided percutaneous needle biopsy of liver, (2) patients with active, enhancing HCC by radiography, (3) patients with PVTT surrounding near the HCC, not located at the distant, separate parenchyma, (4) patients not showing extrahepatic metastases, (5) patients with tumor size (maximal diameter) ≤ 5 cm, (6) age < 75, (7) patients with HCC that did not develop within the transplanted liver, (8) patients who had ECOG score ≤ 3, (9) patients with no previous experience of radiotherapy and (10) patients with leukocytes ≥ 4,000/μ*l*, platelet ≥ 50,000/μ*l*. Written informed consent was obtained from all patients before therapy.

### Treatment and dose prescription

In terms of previous treatment before SBRT, 17 of our patients had received TACE, 3 patients PEI, 6 patients RFA, and 5 patients had not received any previous treatment. All of these patients were those who needed another treatment modality such as conventional RT or SBRT because they, despite previous treatment, were either experiencing progression due to treatment failure or had refused further treatment due to poor tolerable state. TACE to gain a synergistic local effect and to visualize the location of the target. After the placement of a catheter, an emulsion of 10–20 mg adriamycin in 3–10 ml lipiodol was infused into the artery that supplied the tumor. Then, embolization was performed with a variable amount of gelfoam depending upon the tumor size. TACE (range: 1 – 4 times, median: 2 times) was performed after SBRT in patients with advanced HCCs with PVTT, whereas the patients in small HCC were treated with SBRT alone. The interval between TACE and SBRT was at least 4 weeks.

SBRT was administered using the Cyberknife image guided radiosurgery system. The treatment was planned to enclose the planning target volume (PTV) by the 70–85% (median, 80%) isodose line as the prescribed dose. Gross tumor volume (GTV) is defined as the tumor volume, which enhanced contrast at computer tomography (CT) scan. The PTV is defined as a 5 mm margin around the GTV. The total doses administered were 30–39 Gy (median, 36 Gy) at PTV with the prescription isodose level range of 70–85% (median, 80%) in 3 fractions over three consecutive days. The total treatment time per fraction was 2–2.5 hours and the inter-fraction interval was 24 hours at least.

### SBRT procedure and breath-holding technique

Frameless SBRT was carried out at our institution using the Cyberknife SBRT system. Liver parenchyma around the tumor was implanted with four gold markers, which acted as radiographic landmarks for the image guidance system. In image-guided SBRT, the tumor position during treatment is always defined relative to the abdominal CT. The CT image was taken when breathing from the patient reached the maximum expiration. Treatment was delivered in the step, image and shoot sequence. First, the robot positioned the linear accelerator at a fixed beam-pointing position. Then, the patient took a breath and held it in exhalation while the imaging system acquired the targeting data. The patient then took a resting breath, followed by an RT breath-hold in exhalation, during which the treatment beam was turned on. Anywhere from 10 to 50 monitor units of RT were delivered at each beam position, broken up into breath-holding periods of 10–15 sec, depending on the pulmonary capacity of the patient. Once the complete dose for a particular beam direction had been delivered, the robot advanced the LINAC to the next beam position and the imaging/treatment cycle was repeated. The beam pointing during each RT breath-hold in exhalation was based on the tumor position observed during the most recent prior imaging during a breath-hold in exhalation. And the reproducibility of breath hold assessed was checked by skin marker and correlated, matched dot drawn on the monitor at the control room.

### Dose limitation to normal tissues

The liver, stomach, duodenum, intestine, kidney, and spinal cords were contoured during the planning process and dose-volume histograms (DVH) were used to ensure that normal tissue tolerances were not exceeded.

### Liver

Doses of 30–35 Gy in the whole liver with conventional fractionation are often considered to be the limit of liver tolerance. Kazunari Yamada et al. reported that the volume of the liver receiving a dose in excess of 30 Gy, with conventional fractionation, could be used as a predictive test for damage in liver function [19]. We evaluated V20 as a predictor for liver damage accrued from the SBRT in our study: in the α/β ratio of 3, 30 – 35 Gy with conventional fractionation is equivalent to a dose of 3 × 6 Gy (total, 18 Gy) in the whole liver. The V20 was limited so as not to exceed 50% of the functional whole liver tissue.

### Stomach, duodenum and intestine

Due to the lack of clinical data on the effect of very high fraction doses exceeding 8 Gy, the dose of 7 Gy was chosen based on the experience in brachytherapy [20]. Therefore, the maximum dose to the stomach, duodenum, small or large bowel was limited to below 7 Gy per fraction (total, 21 Gy) to avoid serious side effects.

### Kidney

Emani et al. suggested 23 Gy for TD_5/5 _for whole-kidney irradiation [21]. Cassady reported that a threshold dose of 15 Gy delivered with conventional fractionation appeared reasonable [22]. As renal toxicities are usually related to the total volume of treated kidney, DVH are essential to predict renal toxicities. In this study, at least 2/3 of the right kidney was limited to receive a dose of less than 5 Gy per fraction (total, 15 Gy): in the α/β ratio of 3, 23 Gy with conventional fractionation is equivalent to a dose of 3 × 5 Gy (total 15 Gy).

### Spinal cord

The maximum dose to the spinal cord was limited to below the 7 Gy per fraction from the linear-quadratic formula of Withers et al: for an α/β ratio of 3, 42 Gy with conventional fractionation is equivalent to a dose of 3 × 7 Gy (total, 21 Gy) [23].

### Response and toxicity evaluation

Patients underwent abdominal CT scans 1 month after completion of SBRT, and then tumor response was checked at 2–3 month intervals. Tumor responses were classified according to modification of the World Health Organization response evaluation criteria, as follows. Complete response (CR) was defined as complete disappearance of the irradiated tumor, partial response (PR) corresponded to more than 50% reduction in tumor volume, stable disease (SD) was defined as a decrease less than 50% or more than 25% in tumor volume, and progressive disease (PD) as more than 25% increase in tumor volume. CR and PR were defined as objective response.

For the 9 patients with advanced HCC with PVTT, final responses were analyzed after combined modality, SBRT and TACE were performed. Toxicity was evaluated according to the National Cancer Institute Common Toxicity Criteria for Events Version 2.0.

Analysis was performed after 31 patients had been enrolled. The survival time was measured as the period from the date of first SBRT to the date of death or the last follow-up. The survival rate was calculated according to the Kaplan-Meier method using SAS for window 8e.

## Results

### Patients characteristics

Pretreatment characteristics of patients and tumors are summarized in Table 1. The median age was 59 years (range, 44–74 years), and males were predominant. The general condition of most patients was good, with 24 patients having ECOG scores of 0–1. The median target volume was 25.2 cc (range, 3.6–57.3 cc), 23.5 cc (range, 3.6–57.3 cc) in small HCC and 32.8 cc (range: 3.9–47.7 cc) in PVTT. The median follow-up for 31 patients was 10.5 months (range, 2.0–18.5 months), 11.5 months (range, 2.0–18.5 months) in small HCC and 8.5 months (range, 2.0–15.0 months) in PVTT. The summary of SBRT of patients is shown in Table 2.

**Table 1:** Patient characteristics

Characteristics	Small HCC	PVTT	Total
Number of patients	22	9	31
Number of lesions	23	9	32
Age (years)			
Range	44 ~ 78	44 ~ 71	44 ~ 78
Median	60	57	59
Gender			
Male	14	5	19
Female	8	4	12
Tumor volume (cc)			
Range	3.6 ~ 57.3	3.9 ~ 47.7	3.6 ~ 57.3
Median	23.5	32.8	25.2
ECOG performance status			
0–1	18	6	24
2	4	3	7
Child-Pugh classification			
A	19	7	26
B	3	2	5
AJCC stage			
I	8	0	8
II	14	0	14
IIIA	0	9	9

ECOG, Eastern Cooperative Oncology Group, AJCC, American Joint Committee on Cancer

**Table 2:** Summary of primary HCC treated SBRT with or without transarterial chemoembolization

No	Age	Sex	Tumor site	Tumor volume (cc)	SBRT dose (Gy)	Response	Status (months)
1	59	F	Right lobe	25.0	30	PR	DOI(2.0)
2	63	F	Right lobe	5.4	36	CR	NED(18.5)
3	52	F	Right lobe	28.6	30	PR	A&D(16.0)
4	57	F	Right lobe	23.5	36	CR	NED(14.5)
5	78	M	Right lobe	26.5	36	CR	NED(14.0)
6	56	F	Right lobe	31.9	36	PR	A&D(13.0)
7	75	M	Right lobe	25.2/27.5	39/36	SD/CR	A&D(13.0)
8	60	M	Right lobe	36.5	36	PR	A&D(12.5)
9	56	M	Left lobe	15.4	36	CR	NED(12.5)
10	70	F	Right lobe	16.9	36	SD	A&D(12.0)
11	44	M	Right lobe	16.6	30	PR	NED(12.0)
12	48	M	Right lobe	35.1	30	SD	A&D(12.0)
13	77	M	Right lobe	33.3	33	PR	DOI(11.5)
14	60	M	Right lobe	6.9	36	CR	A&D(11.0)
15	55	M	Right lobe	12.0	36	PR	A&D(10.0)
16	62	F	Right lobe	31.3	30	PR	A&D(9.0)
17	56	F	Right lobe	17.6	33	PR	A&D(9.0)
18	62	M	Right lobe	21.6	30	SD	A&D(8.5)
19	75	M	Right lobe	13.6	33	PR	A&D(8.5)
20	48	M	Right lobe	12.0	36	PR	A&D(8.0)
21	62	M	Right lobe	57.3	30	PR	A&D(7.5)
22	67	M	Right lobe	3.6	30	PR	A&D(6.0)
23	57	M	Portal vein	47.7	36	PR	DOM(2.0)
24	60	M	Portal vein	16.2	36	PR	NED(15.0)
25	46	F	Portal vein	28.3	36	SD	DOI(3.0)
26	71	F	Portal vein	3.9	30	SD	A&D(13.0)
27	44	M	Portal vein	38.9	30	SD	DOI(12.0)
28	63	F	Portal vein	34.7	30	SD	A&D(8.5)
29	55	M	Portal vein	37.0	30	CR	NED(8.0)
30	48	M	Portal vein	23.7	30	SD	DOI(7.0)
31	57	F	Portal vein	32.8	30	PR	A&D(6.0)

No, number, SBRT, stereotactic body radiation therapy, CR, complete response, PR, partial response, SD, stable disease, PD, progression disease, DOI, death of intercurrent disease, NED, no evidence of disease A&D, alive of disease, DOM, death of metastatic disease.

### Tumor response and survival to treatments

**Figure 1:**
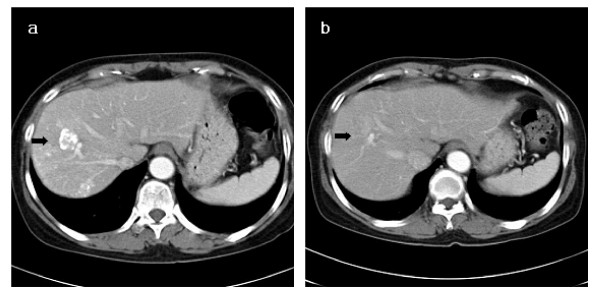
**(a) The initial abdominal CT scan with the primary HCC indicated by the arrow**. (b) CT scan seven months after SBRT. This patient was classified as PR at 5 months after SBRT. (SBRT, stereotactic body radiation therapy).

The overall response rate (CR plus PR) was 71.9% (23/32) [small HCC: 82.6% (19/23), advanced HCC with PVTT: 44.4% (4/9)]. Of the 32 lesions, 7 (21.9%) had a CR [small HCC – 26.1% (6/23), advanced HCC with PVTT – 11.1% (1/9)] and 16 (50.0%) a PR [small HCC – 56.5% (13/23), advanced HCC with PVTT – 33.3% (3/9)]. SD disease was documented in 9 (28.1%) lesions [small HCC: 17.4% (4/23), advanced HCC with PVTT: 55.6% (5/9)] (Table 3). Figure 1, 2 shows the result for patients who were classified as PRs. The median survival of all patients was 11.5 months, with 1-year survival rate of 81.4%. Among patients of small HCC, the median survival time was 12 months and the 1-year survival rate was 88.1%. The median survival time and 1-year survival rate for advanced HCC with PVTT was 8 months and 43.2% (Figure 3). Local recurrence was observed in 3 lesions, observed in small HCC (1) and advanced HCC with PVTT (2). At the time of analysis, 6 patients had died of disease and 25 patients were alive.

**Table 3:** Response of primary HCC treated SBRT with or without transarterial chemoembolization

Type of response	Small HCC No. of lesions(%)	PVTT No. of lesions(%)	Total No. of lesions(%)
Complete response	6 (26.1)	1 (11.1)	7 (21.9)
Partial response	13(56.5)	3 (33.3)	16 (50.0)
Stable disease	4 (17.4)	5 (55.6)	9 (28.1)

**Figure 2:**
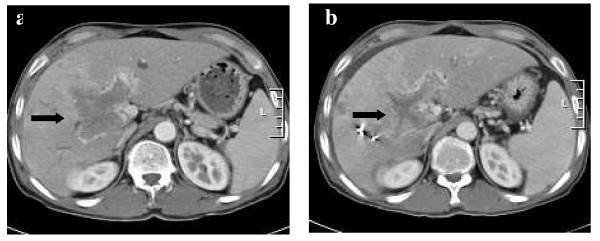
**(a) The initial abdominal CT scan with the PVTT indicated by the arrow**. (b) CT scan two months after SBRT and three courses course of TACE. This patients was classified as PR at 2 months after SBRT. (SBRT, stereotactic body radiation therapy).

**Figure 3:**
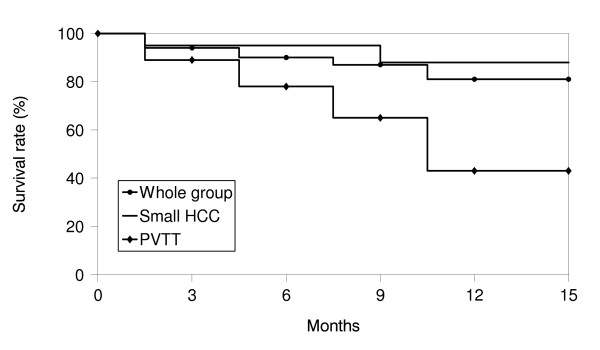
Actuarial overall survival curve.

### Toxicity

The frequency of treatment related toxicities including liver enzymes, bilirubin, albumin, leukocytes, platelets, and nausea toxicities is presented in Table 4. Of the 31 patients, one patient experienced grade 3 liver enzymes toxicity in small HCC. There were no treatment related deaths.

**Table 4:** Toxicity of primary HCC treated SBRT with or without transarterial chemoembolization

Toxicity	Small HCC	PVTT
Liver enzymes, grade		
0	14	4
1	7	5
2	0	0
3	1	0
4	0	0
Bilirubin, grade		
0	18	7
1	4	2
2	0	0
3	0	0
4	0	0
Albumin, grade		
0	19	7
1	3	2
2	0	0
3	0	0
4	0	0
Leukocytes, grade		
0	17	6
1	5	1
2	0	2
3	0	0
4	0	0
Platelets, grade		
0	19	5
1	3	3
2	0	1
3	0	0
4	0	0
Nausea, grade		
0	11	3
1	10	6
2	1	0
3	0	0
4	0	0

Five patients (16.1%) showed progression of Child-Pugh classification from A to B within 3 months after SBRT (3 small HCC, 2 advanced HCC with PVTT). Three of them had progressive disease in 1–2 months after SBRT. Progression from Child-Pugh classification B after SBRT was not observed.

## Discussion

Surgical resection has been considered a preferred modality of treatment for long-term control of some limited HCCs. However, less than 20% of patients are surgical candidates at diagnosis. In recent years, a number of alternative local modalities including PEI, TACE and RFA have been developed. These local treatments have shown local control rates of 86% after PEI [4], local control rates for liver metastasis of 70% after RFA [5] and 55% after TACE [6]. Seong JS et al. studied the combination of conformal RT with TACE for HCC and local control rates of 66% have been reported in patients [24]. As a result of our study, the local control rate was observed to be 71.9%, which is at least equivalent to the invasive local therapies. Therefore, considering the quality of life during and following treatment and the noninvasive, painless approach associated with SBRT, this technique may be a preferred treatment modality for primary HCC.

Clinically, the biologic advantage of a larger volume of potential normal tissue repair, such as that occurs with conventional RT, is of particular importance when the safety margin is small between tumor and normal tissue. If the irradiated volume is restricted to the tumor with a very small security margin, sublethal damage repair is not a first-line aim because complete cell damage is intended. SBRT can create a high-gradient dose falloff in the target tumor with a very small security margin.

Dose escalation appears to be a very important issue for local control rates. If HCC is treated with RT alone, it requires normal liver tissue-sparing radiation techniques, because the tolerance dose of the liver declines with the volume irradiated [25]. However, dose escalation with conventional RT is limited by prolonged treatment time (accelerated tumor cell repopulation) and increase of the dose to the functional liver tissue (impairment of liver function). SBRT can deliver a high dose of radiation to the target tissue with a high degree of precision within the body [26], while sparing most of the adjacent organ, resulting in potentially better local control and lower risk for RT toxicity.

Published clinical data on SBRT, especially for liver tumors, is limited [12-16]. Blomgren et al. published the first experiences of use of stereotactic RT for liver tumors [27]. They recommend a hypofractionated RT approach with an inhomogeneous dose distribution in the target. Herfarth KK et al. used a stereotactic single dose RT approach in the treatment of liver tumors [12]. In these studies, both demonstrated high local tumor control rate and low morbidity. The present study on SBRT was attempted based on the results from the above researches, and the results of this study confirmed that SBRT is indeed helpful in the patients of small HCC.

The presence of PVTT is an extremely poor prognostic factor, because it can lead not only to, the wide dissemination of tumors throughout the liver due to the presence of arterioportal shunting, but also to marked worsening of the liver function as a result of decreased portal flow. In patients with PVTT, TACE was considered a contraindication because it could theoretically result in hepatic damage resulting from hepatic ischemia [28]. TACE is less effective in patient with PVTT, and local RT may make TACE more effective if portal vein disease can be eradicated [29]. Tazawa et al. reported a retrospective study of combined therapy (local RT + TACE) in 24 patients with PVTT [30]. In their study, the survival rate was significantly better in the responders than the non-responders. However, Yamada et al. reported 19 patients who had combined therapy (local RT + TACE) with PVTT; They found no significant difference in overall survival between the responders and non-responders [19]. However, one significant finding in this study was that on follow-up angiograms, the protrusion of PVTT into the main portal trunk decreased in all cases. Combined therapy (local RT + TACE) may prevent PVTT from spreading to the main trunk, suggesting a further benefit of TACE. Because the time frame for the RT period in this study was at least 6 weeks, in many cases, the tumor outside the RT fields continued to be enlarged after RT. A shorter fractionation schedule of SBRT like our study will be able to resolve this problem. Our policy, in combining RT with TACE, was to use SBRT solely to treat PVTT in a short treatment period, whereas intralobar HCC was treated with TACE. SBRT, for primary HCC with PVTT, has shown acceptable local control.

In our study, we observed three patients with local recurrence in tumor of left hepatic lobe of the liver. All of the local recurrence are regarded to resulting from marginal recurrence due to inaccurate treatment of a moving target and the poor breathing capacity of the patients. The magnitude of respiration-induced target motion could be as large as 2–3 cm, peak-to-peak. Various methods have been proposed to control or mitigate target motion. These include active or passive breath-holding techniques [31], respiratory gating [32] and 4 dimension or tumor tracking [33]. Breath-holding techniques, by either actively or passively suspending the patient's respiration, allow treatment during this interval. A study examining intra- and interfraction reproducibility of diaphragm position within a fraction can be reproduced satisfactorily. However, daily imaging and repositioning are still required in order to achieve any appreciable reduction in treatment margin for precise treatment. Meanwhile, we suggest to select future patients with well tolerable breathing or train patient to maintain stable breathing, in order to avoid inaccurate treatment of a moving target.

## Conclusion

Our study shows that SBRT for small HCC and a combination of SBRT with TACE for advanced HCC with PVTT are feasible and effective treatments. Further study is necessary to define the role of dose administered as well as fractionation and side effects in selected patients with small primary HCC and advanced HCC with PVTT.

## Competing interests

The authors declare that they have no competing interests.

## Authors' contributions

BOC, IBC, HSJ, YNK, JSJ, SHB, SKY, GYC and KMK have made substantial contributions to conception and design of the study. BOC, IBC, HSJ, SHB, SKY and KMK carried out acquisitions of data. BOC, IBC, HSJ, YNK, JSJ, GYC and KMK carried out analysis and interpretation of data. BOC, IBC, HSJ, GYC and KMK have been involved in drafting the manuscript.

## Pre-publication history

The pre-publication history for this paper can be accessed here:


